# Screening of rice drought-tolerant lines by introducing a new composite selection index and competitive with multivariate methods

**DOI:** 10.1038/s41598-022-06123-9

**Published:** 2022-02-09

**Authors:** Atefeh Sabouri, Ahmad Reza Dadras, Matin Azari, Abbas Saberi Kouchesfahani, Mehraneh Taslimi, Reza Jalalifar

**Affiliations:** 1 Department of Agronomy and Plant Breeding, Faculty of Agricultural Sciences, University of Guilan, P. O. Box: 41635-1314, Rasht, Iran; 2 Crop and Horticultural Science Research Department, Zanjan Agricultural Resources Research and Education Center, Agricultural Research, Education and Extension Organization (AREEO), Zanjan, Iran

**Keywords:** Plant sciences, Plant breeding

## Abstract

Selection and breeding for drought tolerance in rice have always been one of the leading objectives for rice breeders in water-deficient farming areas. In the present study, we applied the potential of recombinant inbred lines (RILs) population, which were derived from cross Shahpasand (Iranian landrace) and IR28, for the development of drought-tolerant rice lines. One hundred fifty-two lines along with five check varieties were investigated from 2017 to 2019 under non-stress and drought stress conditions. The yield reduction caused by drought based on overall mean during 2017, 2018, and 2019 were estimated to be 89.40, 57.95, and 35.31%, respectively. Using different statistical methods, certain lines, including L33, L90, and L109, which are considered as the best lines in most environments, were found to be promising for being utilized to increase rice drought tolerance. The averages of grain yield of the above-mentioned lines were respectively 6.45, 5.80, and 5.70 t ha^−1^ under non-stress condition, and respectively 2.77, 2.66, and 2.59 t ha^−1^ under drought stress condition. The yield reduction of the selected lines were significantly lower than that of others indicating the significant transgressive segregation. The results revealed using the combination of the best identified tolerance and susceptibility indices and GT-biplot are effective methods for screening superior lines. However, their utilization is not easy and requires specialized packages. For the first time, we introduced a new composite index as a combination of significant indices (CSI). CSI is in the form of a linear function of indices which effectiveness is determined by their correlation coefficient with grain yield. According to our results, using CSI, the identified rice drought-tolerant lines were in high agreement with those obtained by other methods, demonstrating that CSI is a simple but reliable composite index.

## Introduction

Rice (*Oryza sativa* L.) is a major staple food for most people living in Asia and developing countries. Owing to its phylogenetic origin as a semi-aquatic plant^1^, rice is highly dependent on a sufficient amount of water for growing. Due to the increasing temperature and decreasing precipitation, drought has been one of the most important limiting factors for crop productivity and, eventually, for food security worldwide. Global warming and unpredictable rainfall patterns in recent years have led to excessive drought spells causing large yield losses and excessive scarcity in food production in numerous parts of the world^2^. A meta-analysis has revealed that rice yield has declined in recent years in view of drought, and that future droughts might result into even lower yields of rice compared to the current drought^3^. Therefore, the identification and development of appropriate drought-tolerant rice genotypes, is one of the prime objectives of plant breeders in dealing with water-deficient problem.

The success of a breeding program depends on the choice of parents and an appropriate selection method. Recombinant inbred lines (RILs), an ideal population for genetic mapping as well as breeding studies, are produced using the single seed descent (SSD) method. SSD can be used to generate a wide range of genetic variation, to increase the likelihood of transgressive segregation and to produce homozygous or near homozygous lines. Another advantage, in contrast to F2 population, is production of sufficient seed for carrying out the experimental designs with replications. SSD together with Rapid Generation Advance (RGA) have been used in many breeding programmes to shorten the breeding cycle by controlling crop growth conditions in a greenhouse. Several released varieties of crops have been developed employing SSD or RGA breeding methods, ranging from grain quality features to salinity tolerance^4^.

Janwan et al.^5^ investigated 271 F7 RILs derived from a cross between KDML105 and CH1, and developed by the SSD method and five check varieties. They identified three lines which were significantly high yielding as compared to the best check variety (CNT1) based on transgressive segregation. Jena et al.^6^ identified cold tolerant breeding lines with higher spikelet fertility (51–81%) than the Geumobyeo as a cold-sensitive temperate japonica parent (7%) and the breeding line IR66160-121-4-4-2 as the cold tolerant parent (73%). The researches at International Rice Research Institute (IRRI) have led to the identification of several drought-tolerant breeding lines, including IR74371-70-1-1 and IR74371-54-1-1, for cultivation in India and Philippines, respectively, in 2009^2^. In addition, IR 74371-46-1-1 developed from the cross Way Rarem/2*IR55419-04 was released as the drought-tolerant variety Sookha Dhan1 for rainfed lowland ecosystem in Nepal in 2011. Kumar et al.^7^ reported that 17 high-yielding drought-tolerant varieties were developed from IRRI’s drought breeding programs, which were released in different countries of South and Southeast Asia and Africa. Conventional and molecular approaches were applied in order to develop these varieties.

However, the development of drought-tolerant varieties has not been fast enough^8^. This could be attributed to the coupling of photosynthesis and transpiration processes when water is limited, incomplete understanding of the mechanisms of drought tolerance, and different stages of drought occurrence. One of the most important reasons is the complex genetic control of drought tolerance and consequently, a large genotype by environment interactions for yield. Accordingly, it is of great necessity to test the performance of the varieties in different years and locations^8^.

Several selection indices, including SSI (Stress susceptibility index^9^), RSI (Relative stress index^10^), TOL (Tolerance^11^), MP (Mean productivity^11^), YSI (Yield stability index^12^), HM (Harmonic mean^13^), GMP (Geometric mean productivity^14^), STI (Stress tolerance index^14^), and YI (Yield index^15^) have been proposed on the basis of the relationship between grain yield under non-stress and stress conditions. However, their selection based on a combination of the best indices utilizing different mathematical methods might be more appropriate for the identification of the superior genotypes under both conditions. Some multivariate analysis such as principle component analysis and cluster analysis could provide a simultaneous analysis of multiple variables to improve the ranking accuracy of the genotypes^16^. These methods have been employed for the identification of superior lines using selection indices^17,18^. Indeed, a combination of indices may provide a more appropriate criterion for selection in breeding programs. Therefore, it is necessary to introduce a general index with the simplicity of calculation and efficiency. We here introduced a new index as a combination of indices (CSI) significantly correlated with grain yield under non-stress and stress conditions. The results of selection by CSI and multivariate analysis were compared, and its efficiency was discussed.

On the other hand, biplots are widely used graphical technique to display the inter-relationships between variables as well as the observations from a multivariate data on the same plot. Multi environment trials typically evaluate sets of genotypes across years and locations for several economically important traits, to predict the performances of genotypes in future trials. The change in response of the genotypes to changing environments is the Genotype-environment interaction (G × E). One of the commonly used models for biplot analysis applied in several plants for G × E studies is the Genotype and Genotype × Environment Interaction (GGE) biplots. GGE-biplots display both genotype (as main effects) and G × E interaction components, which are the two important sources of variation that have to be considered simultaneously for varietal evaluation across environments^19^. In addition, Genotype × trait (GT)-biplots has also been used to identify of traits that are reliable for indirect selection of a target primary trait^20^. Previous studies have demonstrated the use GT-biplots in evaluating genotypes based on various selection indices and the relationship among them^21–23^.

Thus, we developed an RIL population derived from a cross between IR28 and Shahpasand (SH). We evaluated the RILs for 3 years under non-stress and drought stress conditions. We conducted the current research to:

1. Determine the best indices to discriminate the lines based on correlation coefficient with grain yield as well as GT-biplot tester-focused scaling
2. Identify superior lines under drought and non-stress conditions using polygon view of GT-biplot symmetrical scaling based on selection indices in the rice RILs.
3. Identify stable and superior lines using polygon view of GGE-biplot symmetrical scaling considering mean performance and stability of lines during 3 years.
4. Introduce a new composition index (CSI) to screen drought-tolerant lines and to compare its efficiency to other methods.

## Results

### Transgressive segregation

**Figure 1:**
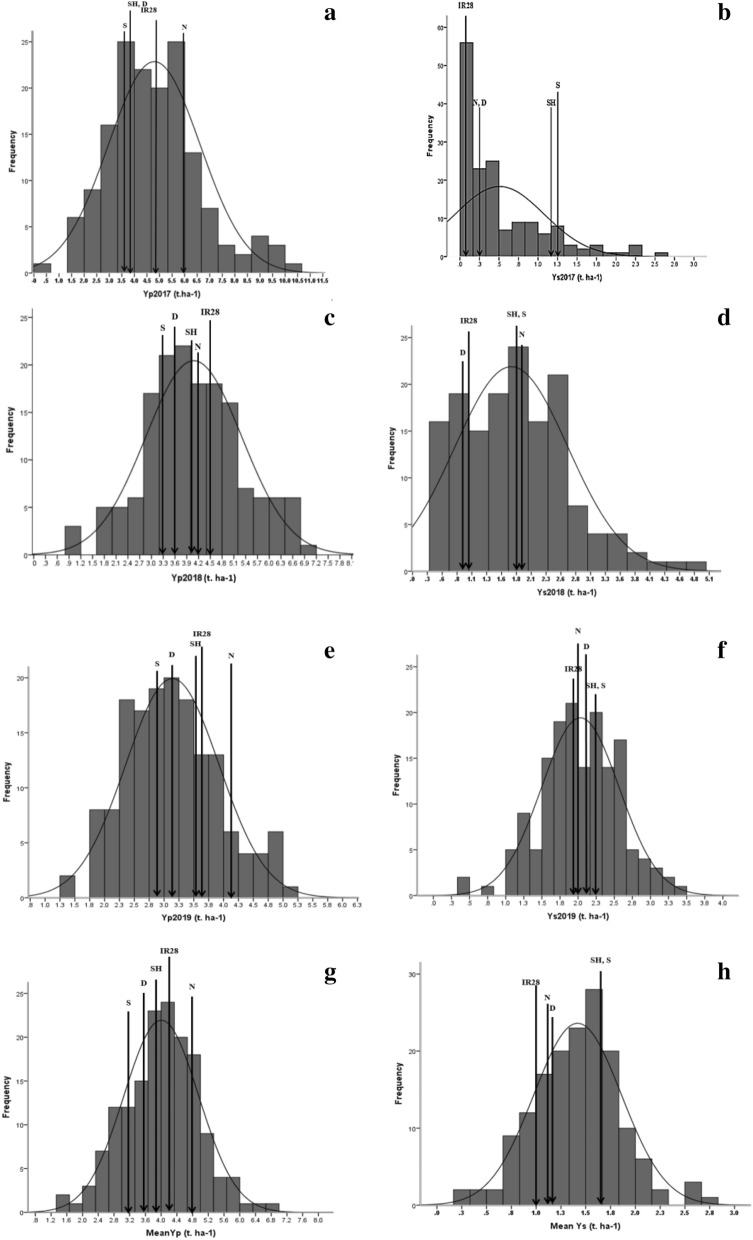
The histogram of frequency distribution of grain yield of 152 rice RILs, parental (IR28 and SH: Shahpasand) and check varieties (N; Neda, S; Sadri, D; Dorfak) under non-stress condition in 2017 (**a**), drought stress condition in 2017 (**b**), non-stress condition in 2018 (**c**), drought stress condition in 2018 (**d**), non-stress condition in 2019 (**e**), drought stress condition in 2019 (**f**), non-stress condition in average of all 3 years from 2017 to 2019 (**g**) and drought stress condition in average of all 3 years from 2017 to 2019 (**h**).

A significant variation and transgressive segregation were observed in the RIL population. Transgressive segregant lines in both directions (positive and negative) revealed higher and lower values than parents and check varieties, respectively, for all six environments (two environments, non-stress and drought stress per year). Figure 1 presents the histogram of frequency distribution of yield under normal and drought stress condition for 3 years along with parental and check varieties. The extreme transgressive segregant lines were as follow:

Lines L90, L33, L109, L32, L124, L144, L59, L45, L35, L31, L53 and L15 were higher and lines L49, L3, L65, L36, L25, L10 and L61 were lower yielding than parents in both non-stress condition and drought stress condition.

The lowest grain yield under drought stress and highest reduction in 2017 were estimated to be 0.503 and 89.50% respectively, which was due to severe drought stress as compared to the other years based on the average of grain yield of the population (Supplementary Table 1). In 2018 and 2019, grain yield reductions were 58.23% and 38.60%, respectively. The highest grain yields of the RILs under non-stress (4.79 t ha^−1^) and drought stress conditions (2.03 t ha^−1^) were obtained in 2017 and 2019, respectively. Generally, the mean yield of the RILs in all environments was in the median of parental and check varieties which referred to the high diversity in the RILs. Also, in all environments due to transgressive segregation, a high range was estimated for yield. Two varieties of Sadri (S) and SH showed more drought tolerance than other varieties (1.79 and 1.83 t ha^−1^ in average of all 3 years, respectively) and IR28 (sensitive parent) was found as the most sensitive variety with 1.04 t ha^−1^ in average of 3 years (Supplementary Table 2).

Significant correlations (*P < *0.001) was found between grain yield and days to flowering under drought stress condition with coefficients − 0.433**, − 0.483**, − 0.491** across 3 years respectively. Lines with developmentally faster (i.e. sooner flowering) have higher grain yield.

Preliminary yield experiments were performed using augmented block design with four replications and five check varieties. Therefore, a combined analysis as RCBD design was conducted and the effects of year, check variety, and check variety × year interaction were studied. The analysis of variance (ANOVA) revealed significant differences (*P* < 0.01) among the year, check variety, and check variety × year interaction regarding grain yield under both conditions (non-stress and drought stress condition) (Table 1). Based on the results of ANOVA in each year individually, the replication effect was not significant.

**Table 1:** Summary of combined analysis of the grain yield (GY) of five check varieties as RCBD design during 3 years with four replication (Rep) under non-stress and drought stress conditions across 3 years 2017–2019.

S.O.V	DF	Mean squares	
		Non-stress	Stress
Year (Y)	2	7.878**	24.354**
Rep/Y	9	0.054	0.049
Check variety (C)	4	5.293**	2.074**
C × Y	8	0.735**	0.912**
Error	36	0.179	0.039
CV		10.01%	10.15%

**Significant at 0.01 probably level by F test.

Considering to significance of check variety × year interaction, data analysis was performed for each year individually, as well as one analysis based on pooled data and the overall mean during 3 years for comparing the results.

The effectiveness of the indices in identifying the best drought-tolerant lines using two methods (correlation among indices with grain yield and GT-bipolt) were compared.

### Identification of the best indices based on the correlation among indices and grain yield

**Figure 2:**
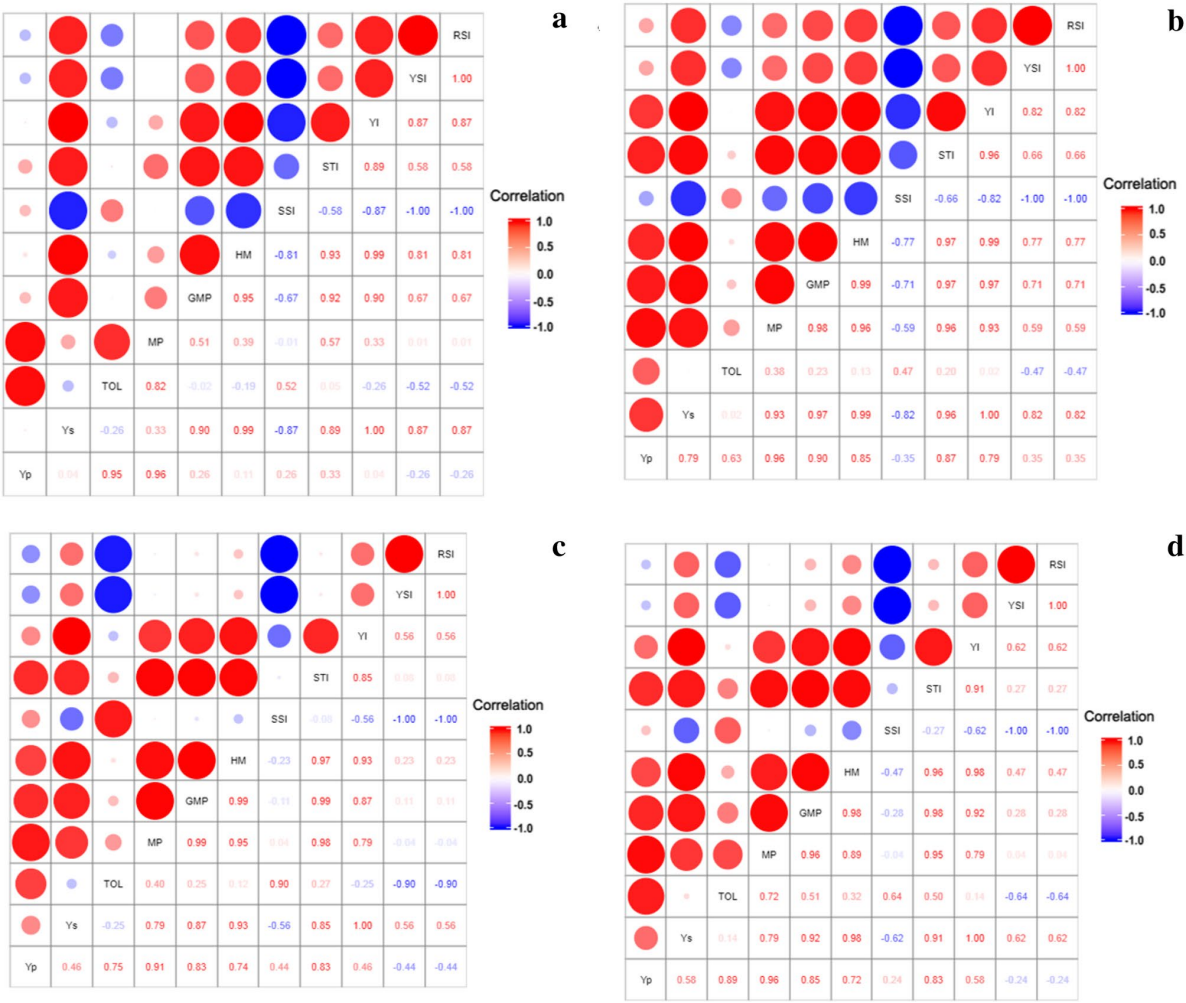
Correlation coefficients and diagram between grain yield under non-stress (YP), and drought stress condition (YS) along with nine indices values for the 152 rice RILs, parental (IR28 and SH: Shahpasand) and check varieties (N; Neda, S; Sadri, D; Dorfak) in 2017 (**a**), 2018 (**b**), 2019 (**c**) and average of all 3 years from 2017 to 2019 (**d**).

Although several significant correlation coefficients (*P* < 0.05 and *P* < 0.01) were obtained, for RSI, YSI, YI, STI, HM, and GMP, we estimated positive and significant correlations with grain yield under drought stress (YS), and negative and significant correlation with SSI. Meanwhile, TOL and MP demonstrated a positive and significant correlation with grain yield under non-stress (YP) in 2017. However, MP, GMP, HM, and STI showed a positive and significant correlation with YP and YS during 2018, 2019, and the overall mean of 3 years. YP had a significantly positive correlation (*P* < 0.01) with MP (r = 0.96), GMP (r = 0.85), HM (r = 0.72), and STI (r = 0.83); YS had a significantly positive relationship (*P* < 0.01) with MP (r = 0.79), GMP (r = 0.92), HM (r = 0.98), and STI (r = 0.91). Positive correlations of MP, GMP, HM and STI with YP and YS indicate that selection based on MP, GMP, HM and STI will result in increased yield under both non-stress and stress conditions. Figure 2 illustrates the correlation coefficients between indices and grain yield in the 3 years and the overall mean under non-stress and drought stress.

**Figure 3:**
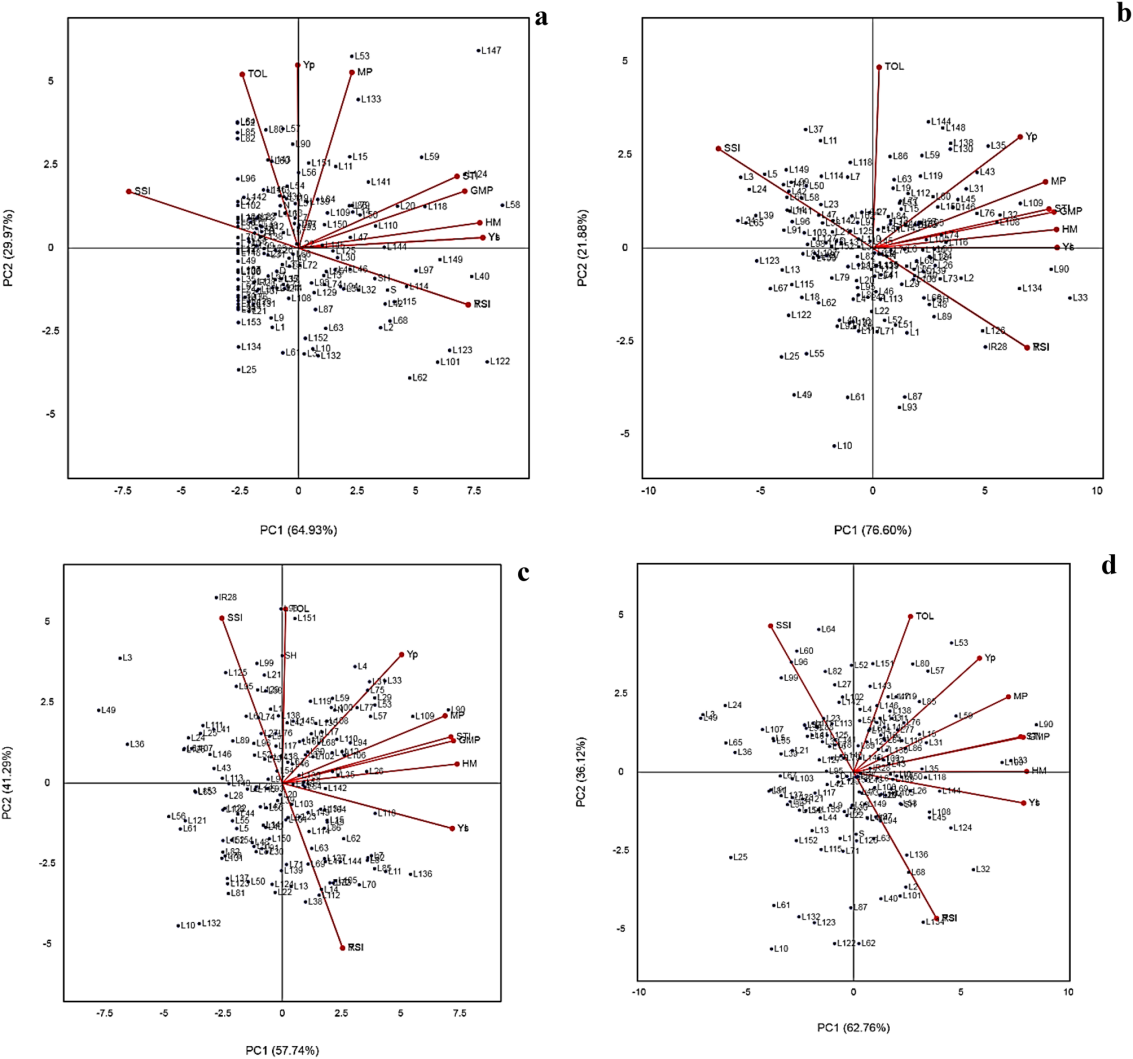
Graphic display biplot for drought tolerance indices based on grain yield of 152 rice RILs, parental (IR28 and SH: Shahpasand) and check varieties (N; Neda, S; Sadri, D; Dorfak) under non-stress and drought stress conditions in 2017 (**a**), 2018 (**b**), 2019 (**c**), and average of all 3 years from 2017 to 2019 (**d**).

Principal component analysis (PCA) to identify the best indices, as well as the best promising lines, showed based on the year-wise and the average yields, the indices best represented by PC1 are YS, MP, GMP, HM, and STI and therefore this component stands for tolerance to drought and are better predictors for yield under stress. Genotypes with high PC1 scores are identified as drought tolerant. PC2 is highly correlated with TOL in all cases and therefore stands for drought susceptibility and genotypes with high PC2 scores are drought susceptible. YP is well represented by both components except for 2017 where it was close to PC2. Thus, MP, GMP, HM, and STI were better predictors of YS and YP than TOL. Therefore, selecting genotypes with high PC1 and PC2 scores had a better performance compared to the other genotypes, parents and the check varieties. PCA on the 2017 data showed that, the first component (PC1) explained 64.93% of the variations and exhibited a significant correlation coefficient among GMP, HM, STI, and YS. The second component (PC2) explained 29.97% of the total variation and revealed positive correlations among TOL, MP, and YP. **L58, L118, L20, L59, L144, L110, L141, L15**, and **L11** were in favor of high PC1, and **L133, L147**, and **L53** were supported by high PC2. **L147** was in favour of both PC1 and PC2 (Fig. 3a). PCA based on 2018, 2019 and the overall mean data were more similar. PC1 explained 76.60%, 57.74%, and 62.76% of the variations, respectively, with significant correlations among YP, YS, MP, GMP, HM, and STI. Additionally, PC2 had a strong correlation with TOL and SSI explaining 21.88%, 41.29%, and 36.12% of the variations, respectively (Fig. 3b–d). The lines **L90, L33, L109, L108, L118, L136, L11, L85, L32,** and **L144** were almost common in the position of biplots in 2018, 2019 and the overall mean.

### Identification of the best indices based on GT-biplots

**Figure 4:**
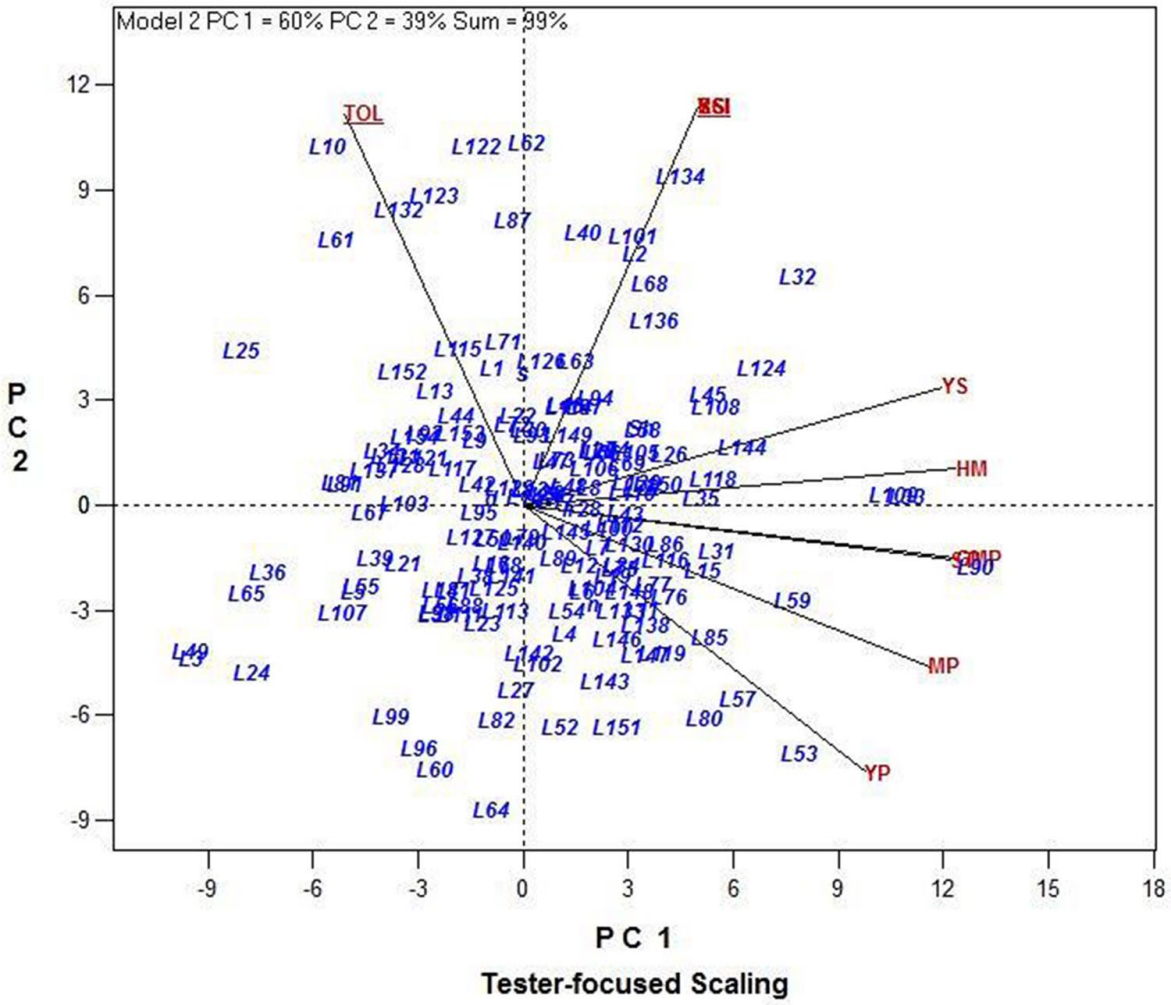
GT-biplot for different drought tolerance indices based on average of grain yield of 152 rice RILs, parental (IR28 and SH: Shahpasand) and check varieties (N; Neda, S; Sadri, D; Dorfak) under non-stress and drought stress conditions during 3 years from 2017 to 2019. The angles less than 90° between the indices and grain yield represent MP, GMP, STI and HM had strong positive relationship with grain yield under non-stress (YP), and drought stress condition (YS).

A GT-biplot tester-focused scaling was generated according to Yan and Rajcan^24^ for different drought tolerance indices, based on the average of grain yield of the population from 2017 to 2019. This type of scaling was used for indices comparison. The cosine of the angle between the indices indicated the correlation between them. These plots showed that MP, GMP, STI, and HM had a strong positive relationship with YP and YS (Fig. 4). The obtained results herein confirmed the above-mentioned findings. Generally, it is evident that the highest common correlation with YP, and YS over the years belonged to the indices, namely MP, GMP, HM, and STI. These indices could be considered helpful for identifying the best performing lines under non-stress and drought stress conditions. Furthermore, we used GT-biplots for determining the most desirable indices in discriminating the lines in each year individually (Supplementary Fig. 1) through the comparison of all the indices with the ideal index. An ideal index should be both discriminating and representative. The small circle on the axis, with an arrow pointing to it, represents the ideal index and is used as a reference point. This ideal index was employed as the center of a set of concentric lines that serve as a ruler to measure the distance between an index and the ideal index. These biplots in Supplementary Fig. 1a–d depicts that GMP and STI were the closest to the ideal index, and therefore, they were the most desirable of all the indices, followed by HM in all the plots.

### Identification of promising lines for drought stress based on polygon view of the GT-biplot

**Figure 5:**
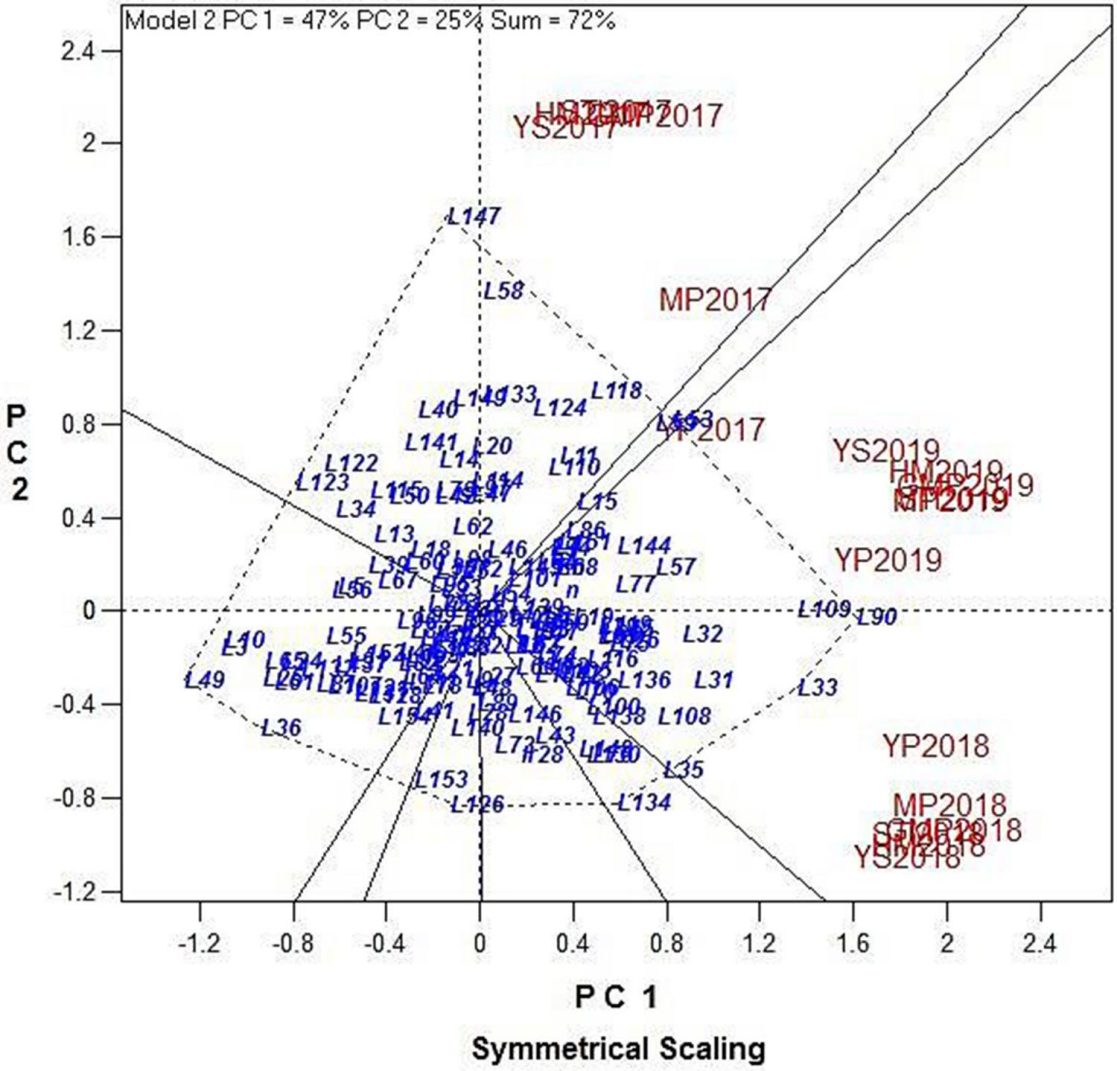
Polygon view of the rice RILs GT-biplot, showing which line had the highest values for which drought indices during 3 years.

A polygon view of the rice RILs GT-biplot with symmetrical scaling considering all the 3 years is presented in Fig. 5. This polygon is drawn by joining the genotypes located away from the biplot origin so that all the other genotypes are contained in the polygon. Perpendicular lines were drawn to each side of the polygon, which divided the biplot into the sectors. The vertex line in each sector exhibits the most efficient line in the environment that falls in that specific sector. Lines **L147, L118, L58,** and **L53** are the best performers in the sector of 2017 indices, and **lines L90, L109, L33, L136,** and **L35 for** the sectors of 2018 and 2019 indices. In contrast, lines **L10, L25, L49, L24,** and **L122** were in the vertex of the polygon, yet not in the vicinity of any of the indices and yield, suggesting that these lines were of the lowest values for the yield and indices.

**Figure 6:**
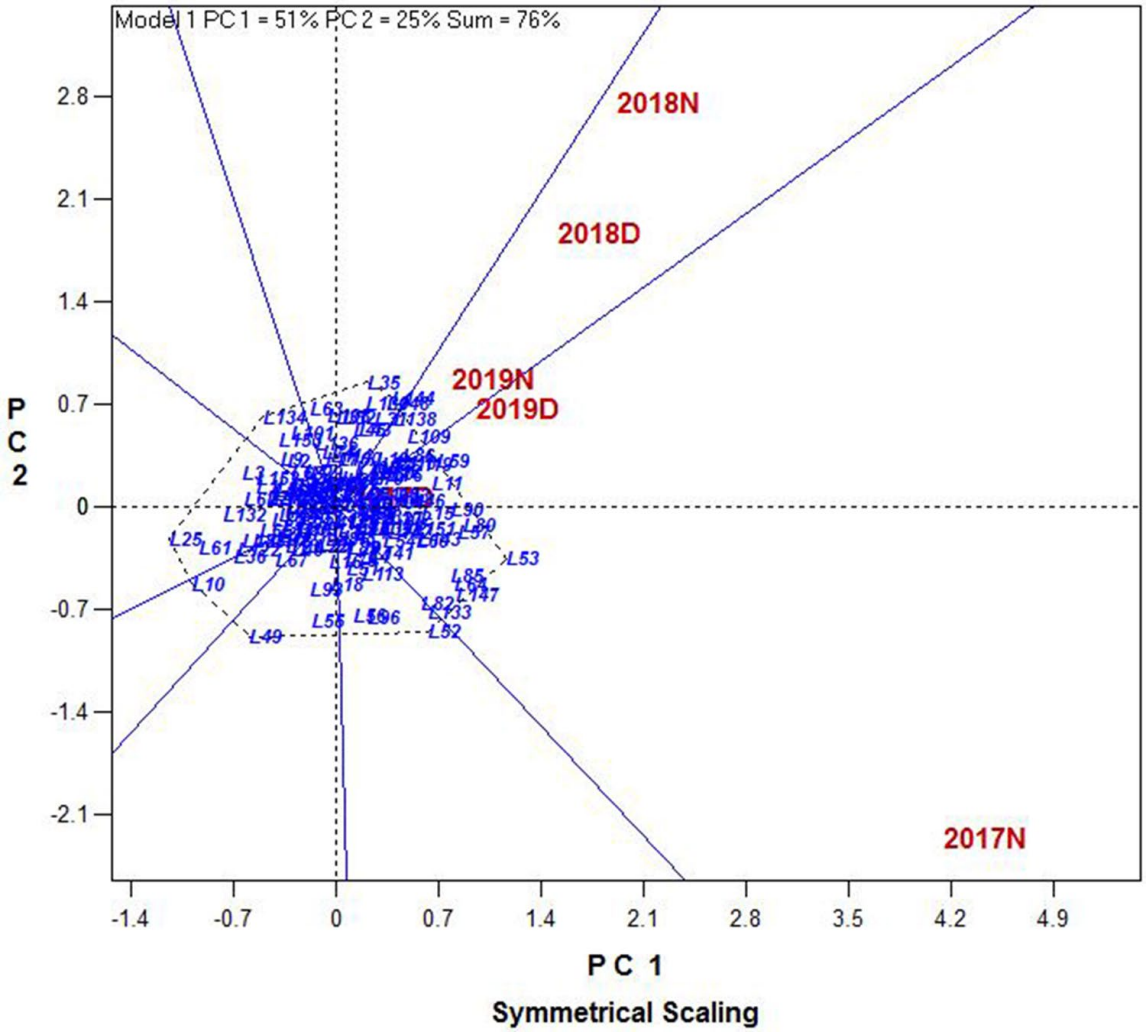
Polygon view of the GGE biplot rice RILs showing which line yielded best in each of six environments including non-stress (N) and drought stress (D) for 2017 to 2019.

Based on ANOVA in both conditions non-stress and drought stress, genotype × year interaction effect was estimated significant. Therefore, it is important to consider the interactions of genotype in the environment and performance of rice lines in these 3 years with different environments. In order to identify the lines that were less affected by the environment and had more stability in the 3 years, we used GGE-biplot with the which-won-where pattern of lines and years. Figure 6 shows a polygon view of the GGE biplot symmetrical scaling rice RILs representing the superior lines in each of the six environments. According to the biplot, the sector of 2017N (non-stress condition in 2017), 2017D (drought stress condition in 2017), and 2019D, lines **L85, L64, L147, L53, L80, L57, L90, L11,** and **L59** produced the highest grain yield. Similarly, in 2018D, 2018N, and 2019N sectors, lines **L109, L86, L19, L119**, and **L76** yielded the highest. Two lines **L11** and **L80** were distinguished by considering GE interaction in the GGE-biplot.

### Identification of promising lines based on new index CSI

**Figure 7:**
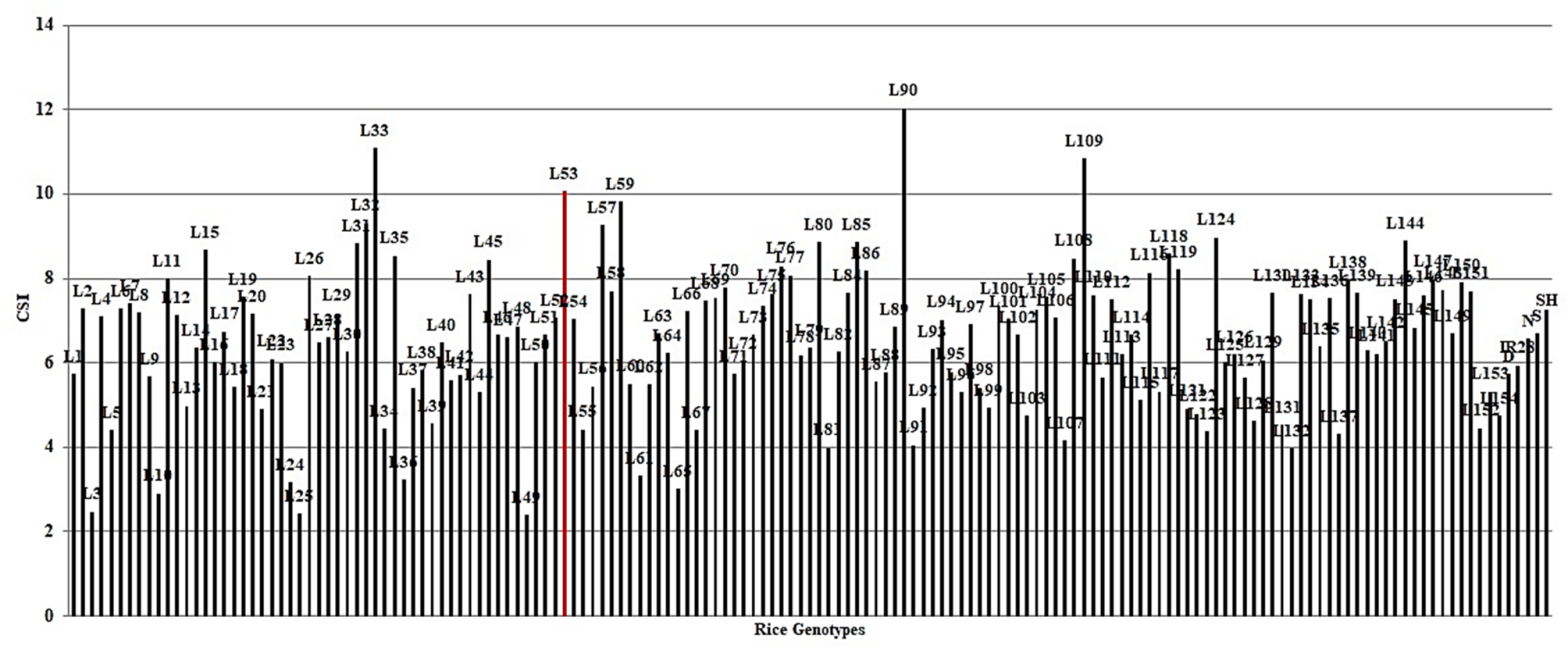
The CSI value for 152 rice RILs, parental (IR28 and SH: Shahpasand) and check varieties (N; Neda, S; Sadri, D; Dorfak) based on overall mean of lines during 3 years (2017–2019).

The new index CSI was calculated for 152 rice RILs, parental (IR28 and SH: Shahpasand) and check varieties (N; Neda, S; Sadri, D; Dorfak) based on indices MP, GMP, HM, and STI in the 3 years and based on pooled data over these years. The highest value of CSI in 2017 belonged to **L147, L58, L124, L53, L59, L133, L118, L40, L20, L149**, and **L141** (Supplementary Fig. 2). Moreover, according to CSI values of rice lines in 2018, **L33, L90, L109, L35, L32, L134, L108, L43** showed the highest performance in both conditions (Supplementary Fig. 3). The best collection of rice lines considering highest CSI value in 2019 comprised **L90, L109, L33, L29, L53, L31, L75, L136, L57, L4, L77,** and **L26** (Supplementary Fig. 4). In addition, Fig. 7 implies that lines L90 and L31 had the highest CSI in the average of all 3 years based on pooled data over 3 years. Supplementary Table 3 presents the raw pooled data with the indices and CSI calculation formula for RILs.

**Figure 8:**
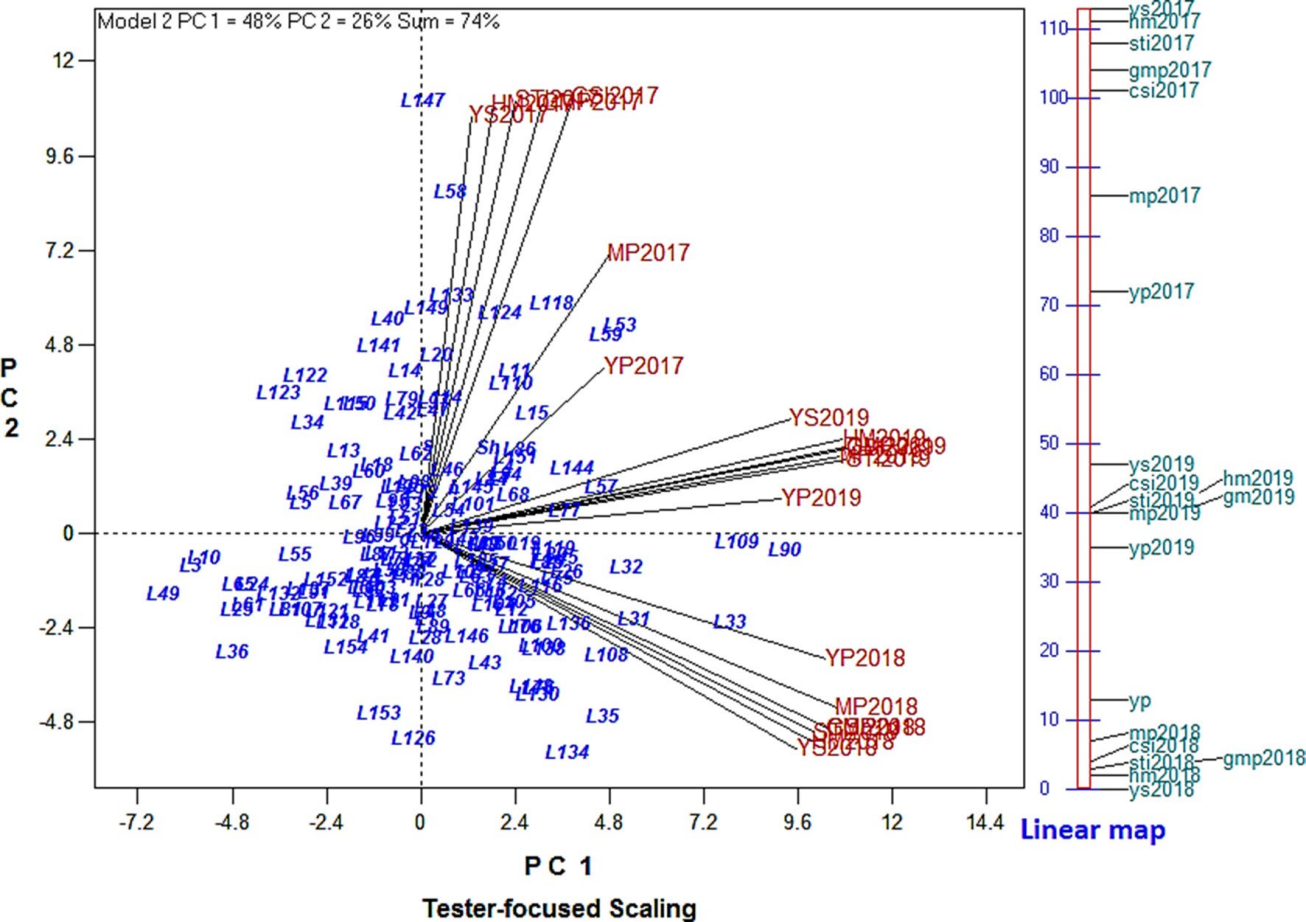
GT-biplot for different drought tolerance indices based on the grain yield data of 152 rice RILs, parental (IR28 and SH: Shahpasand) and check varieties (N; Neda, S; Sadri, D; Dorfak) under non-stress and drought stress conditions in 3 years (2017–2019). The angles less than 90° between the new developed index of CSI and other indices; grain yield under non-stress (YP), and drought stress condition (YS) represent that CSI had strong positive relationship with them. Linear map displays the angles among the vectors of the indices and grain yield.

Interestingly, these results are highly in agreement with those obtained by multivariate methods, such as PC graphic display biplot of indices, and GT-biplot, which were described in previous sections. In order to evaluate of the relationship between CSI and other indices, a GT-biplot tester-focused scaling was generated, based on the data all 3 years (Fig. 8). The angles less than 90° between CSI and other indices, YP and YS, in all 3 years represent that CSI had strong correlations with them.

## Discussion

Yield is clearly the final product of contributions made by several traits that are directly or indirectly associated with grain yield^18^. Grain yield is a complex trait, therefore, from a plant breeder’s point of view, the transgression in grain yield is of considerable significance^25^. The transgressive individuals observed in the RILs have been fixed, and their superiority will be maintained. According to Rieseberg et al.^26^, the frequency of transgression is positively correlated with the genetic divergence of the parental lines. The significant transgressive segregation in the present population could be attributed to the high genetic differences between the two parents. Shahpasand is a traditional Iranian cultivar is of high-quality, aromatic, and moderate maturity, while, IR28 developed in IRRI, is high-yielding, semi-dwarf and resistant to blast disease. The parental survey in the present study showed that the two parents differed significantly for yield under non-stress and stress conditions as well as their tolerance to drought. Grain yield in the rice RILs of the cross between (SH × IR28) clearly indicated a tendency for improvement, and therefore significant transgressive segregation was observed in both directions under non-stress and drought stress conditions.

Due to climate changes and subsequent substantial decline of grain yield caused by drought stress, the need for drought-tolerant crops is increasing. Therefore, improving grain yield under drought stress is an essential breeding objective. Generally, breeding for improved drought tolerance in cereals must be combined with good yield potential^27^. The comparison of grain yield under non-stress and drought stress conditions could be taken into consideration as a desirable index for screening germplasm for tolerance to drought stress^28^. A desirable selection index should allow distinguishing genotypes having high yield under stress and non-stress conditions^29^. High value of MP, GMP, and HM is needed to reach higher grain yield under both non-stress and drought stress condition^29^. Singh et al.^18^ reported that selection of indices including GMP, MP, HM, and STI had a positive and highly significant correlation with bread wheat grain yield under drought stress condition whereas STI, MP, and GMP showed positive significant correlation with grain yield under non-stress condition. They employed GMP, STI, MP, HM, and MRP (mean relative performance) to identify the promising lines. Mariey and Khedr^30^ introduced STI, MP, GMP, and HM as major and suitable indices for selecting barley cultivars with high yield at both environments. Singh et al.^17^ also utilized GMP, HP, MP, and STI as the most suitable indices for the selection of superior tolerant bread wheat genotypes. STI, MP, and GMP were found to be effective selection indices during the identification of rice genotypes for sodic and salinity stress^31^. The obtained findings herein also confirmed that MP, GMP, HM, and STI had the highest common correlation with YP and YS over the years.

We used GT-biplot for selecting the most desirable index in discriminating the lines. GT-biplot can be used for identifying key traits, which is sufficient as a selection criterion to select for yield^32–34^. Badu-Apraku and Akinwale^32^ utilized GT-biplot for the identification of the most reliable traits for selecting maize Striga resistance genotypes. Sharifi and Ebadi^34^ determined the most influencing traits on rice grain yield based on the GT-biplot view. GT-biplot has been used to visualize correlations among traits, assess genotypes based on multiple traits and study genotype by trait relationships. Akçura et al.^22^ has demonstrated the use GT-biplots in evaluating genotypes under drought stress condition based on various selection indices and the relationship among them. Based on the results, GMP, STI, and HM were the closest to the ideal index and were recognized as the most desirable and effective indices over the 3 years. These results were in agreement with correlation coefficients. Among the tolerance indices GMP is geometric mean that is less sensitive to the significant differences between YS and YP; therefore, it is a more efficient index than MP, particularly under severe stress conditions^14,35,36^. STI, proposed by Fernandez^14^, is more efficient than MP and GMP in distinguishing between superior genotypes in both non-stress and stress condition and genotypes with high performance only under non-stress condition^36,37^. In contrast, susceptibility indices, including SSI and TOL, tend to distinguish between the tolerant and the susceptible genotypes^37^. However, White et al.^38^ demonstrated that TOL was not able to distinguish between potentially tolerant genotypes and genotypes with low-yield potential. Similarly, Clarke et al.^28^ showed the same limitation for SSI. Although some of the indices are more successful in the discriminant of desirable genotypes under both non-stress and stress conditions, none of tolerance and susceptibility indices alone are ideal for characterizing tolerant and high-yield genotypes under both conditions^37^. Thus, a combination of indices might provide a more appropriate criterion for selection in breeding programs. The proposed index CSI provides selection based on a combination of the best indices, and has some practical advantages as follows:

1. Similar to multivariate methods, CSI considers several indices simultaneously, but the calculation of CSI is easier and simpler and does not require specialized statistics software packages.
2. In the linear function of CSI, the contribution of each index is determined by its coefficient correlation with YP and YS. Therefore, the same contribution is not considered for different indices.

Significant agreement of the results of CSI with the results of PC graphic display biplot, and GT-biplot confirmed the CSI could be considered helpful for identifying the superior genotypes under stress and drought stress conditions easier and faster.

The average rainfall in the growing season of 2017 was lower (Supplementary Table 1), and the lines faced severe drought stress than in 2018 and 2019. Therefore, there were differences between the results of data analysis in 2017 compared to 2018 and 2019 (Fig. 1). Several rice lines produced low yield due to severe drought stress (Fig. 1-b). Based on the results of different method including GT-biplot and CSI index lines **L147, L58, L124, L53, L59, L133** and **L118** in 2017 (with average grain yield 7.65 and 1.81 t ha^−1^ under non-stress and drought stress condition respectively) and lines **L90, L33, L109, L108, L118, L136, L11, L85, L32,** and **L144** showed the highest performance in both conditions in 2018 and 2019. Considering the results of PC-biplot, GT-biplot, and CSI index, certain lines demonstrated a higher performance in each environment. Among the best-identified lines, some lines, including **L33, L90, L109, L53,** and **L58**, were found to be the best lines in several environments. They might also be called the most drought-tolerant lines during the 3 years. According to the results of GGE-biplot lines **L109, L59, L11, L90, L80** and **L53** were distinguished as stable and superior lines under both non-stress and drought stress conditions considering the grain yield of lines during these 3 years and **GE** interaction. The average grain yield of the above-mentioned lines under non-stress and drought stress condition were 5.94 and 2.11 t ha^−1^, respectively. The total mean of RILs with check varieties under non-stress and drought stress condition during the 3 years were estimated as 4.00 and 1.41 t ha^−1^ respectively. The averages of grain yield of **L33, L90,** and **L109** were respectively 6.45, 5.80, and 5.70 t ha^−1^ under non-stress condition, and respectively 2.77, 2.66, and 2.59 t ha^−1^ under drought stress condition.

There was significant negative correlation between grain yield and days to flowering under drought stress condition across 3 years indicating that lines with developmentally faster (i.e. sooner flowering) have higher grain yield. These results are in agreement with findings of Prince et al.^39^ and Kang et al.^40^ which showed days to flowering was negatively correlated with grain yield under drought stress condition.

Akinwale et al.^20^ stated that in comparison with other multivariate analysis methods, GGE biplot has the widest applicability in the analysis of plant breeding data. The lines with higher stability can be detected with GGE biplot. In our work, **L11** and **L80** in GGE biplot were more evident than analysis of each year separately. However, we should bear in mind that the results of a GGE biplot can be deficient if the PC1 and PC2 account for only a small proportion of the total GGE. This happens once the main effect of the genotype is considerably smaller than that of the G × E interaction^20^. In the present study, PC1 and PC2 for plot yield in 3 years (Fig. 6) were estimated as a total of 76% (51 and 25% respectively), which indicates that the interpretation of GGE biplot analysis is reliable.

To solve the issue of water scarcity developing drought-tolerant rice varieties has been a major objective of plant breeders. Moreover, utilizing superior genotypes as donor parents in hybridization could be very effective on developing rice tolerant cultivars.

## Conclusion

The genetic stability of recombinant inbred lines makes it possible to use transgressive segregation in a positive direction. In the present RILs, we observed a significant transgressive segregation for grain yield under non-stress as well as drought stress conditions. Therefore, selection in this population would be useful. Selection indices are effective tools for the identification of superior genotypes under both non-stress and stress conditions and using multivariate analysis makes it possible to make the best use of the combination of useful different informative indices. However, they are complicated and need specialized statistical packages. We found that the use of CSI is easier and requires no specialized software program. Moreover, according to our results, it showed similar effectiveness. The evidence presented in this paper suggests that CSI may provide a suitable criterion for screening in breeding programs. According to the results of the present study, lines **L90, L33, L109, L147, L35, L118, L58, L136, L11** and **L80** has been identified as the most drought-tolerant lines. This suggests that the identified lines may be utilized for further breeding programs against drought tolerance of rice.

## Material and methods

### Plant material and field experiment

All methods were performed in accordance with the relevant guidelines and legislation. The genetic material involved 152 RILs, derived from a cross between IR28 and SH as male and female parents, respectively. Two parents differed in grain yield and drought tolerance. SH and IR28 was tolerant and sensitive to drought respectively. The RILs was developed by selfing F_1_ plants, advancing the generations from F_2_ to F_6_ produced by self-pollination as SSD, and growing individual plants in the field. We cultivated generation F_7_ at the research field of University of Guilan in Rasht. Afterwards, F_8_ to F_10_ populations were grown for preliminary yield experiment using augmented block design with four replications and five check varieties, including parental cultivars (IR28 and SH) and other three cultivars varieties (N; Neda, S; Sadri, D; Dorfak) at the experimental field of Rice Research Institute of Iran (RRII) from 2017 to 2019 under non-stress and drought stress conditions.

The planting and harvesting processes were similar in all 3 years. The seeds of population were sown in the nursery dated 24, 26, and 25 April 2017, 2018, and 2019 respectively. According to soil fertilizer recommendations of soil science laboratory, we applied 100 kg ha^−1^ of potassium oxide (K_2_O) from source of potassium sulfate, 80 kg ha^−1^ of nitrogen from urea source, and 45 kg ha^−1^ of phosphorus pentoxide (P_2_O_5_) from superphosphate triple source in the field. The 25 to 27-day-old seedlings were transplanted with hand, single plant per hill, with 25 cm spacing between the hills and between the rows dated 21, 21, and 20 May in 2017, 2018 and 2019 respectively. The size of each plot was 4 m^2^ for lines and 6 m^2^ for parental and control cultivars. Data on grain yield (after removing borders) were taken from plots 2.5 and 5 m^2^ for the lines and check varieties, respectively. Weed were controlled by hand weeding twice per season, and pest management measures were carried out when required.

The field experiments were conducted under two conditions: drought stress and non-stress, for 3 years. In the non-stress condition, we employed the traditional submerged cultivation method while for the drought stress experiment field, we discontinued the irrigation from ~ 50 to 55 days after transplanting until the end of plant growth. The stress fields were drained 3 weeks ahead of anthesis as applying drought stress before flowering directly affects grain yield in lowland rice^41^. A canal was excavated in a 50-cm depth all around the drought stress experiment field, prior to irrigation withhold, which could allow water drain out of the field. Furthermore, in order to determine soil water status, the soil water potential was estimated utilizing soil moisture retention curves based on soil water content (%vol) by sampling the soil every week after water withholding in seven stages throughout the 3 years (Supplementary Table 4). The soil texture was identified to be clay-loam close to clay based on the texture determination experiment at the soil science laboratory of RRII. A severe drought stress occurred in 2017 due to the lowest rainfall during the growing season. Supplementary Table 1 represents the meteorological data in Rasht in the cropping season from 2017 to 2019.

### Statistical analysis

To demonstrate transgressive segregation, we utilized SPSS version 22, Chicago, IL, USA^42^ for drawing the histograms of the frequency distribution of the yield of RILs and check varieties under normal and drought stress conditions from 2017 to 2019. Analysis of variance of check (ANOVA) was performed using SAS^43^ software version 9.4. Various tolerance and susceptibility indices, including TOL, MP, GMP, HM, SSI, STI, YI, RSI, and YSI, were calculated (Table 2) for each genotype and each year independently. Also calculation of indices was performed using the overall mean grain yield of genotypes across 3 years. The correlation coefficients among these indices, grain yield in six environments (two environments; non-stress and drought stress per year), and the overall mean under non-stress and drought stress were calculated. For these analyses, we employed iPASTIC^44^ software. The software is available at https://manzik.com/ipastic/index.php.

**Table 2:** The used tolerance and susceptibility indices in this study with their mathematical formulas.

Index	Formula	Description	References
Stress susceptibility index	$SSI=\frac{1-\left(\frac{YS}{YP}\right)}{1-\left(\frac{\overline{Y}S }{\overline{Y}P }\right)}$	Assesses the reduction in yield caused by unfavorable compared with favorable environments. Lower value indicates lower differences in yield across the two stress levels, which means more tolerant to drought	Fischer and Maurer^9^
Relative stress index	$RSI=\frac{(\frac{YS}{YP})}{(\frac{\overline{Y}S }{\overline{Y}P })}$	The genotypes with high RSI value will be more tolerant	Fischer and Wood^10^
Tolerance	$TOL=YP-YS$	Higher values of TOL indicate susceptibility of a given genotype	Rosielle and Hamblin^11^
Mean productivity	$MP=\frac{YP+YS}{2}$	Higher value of MP indicates higher rate of productivity	Rosielle and Hamblin^11^
Yield stability index	$YSI=\frac{YS}{YP}$	Higher value of YSI indicates more stability of the genotype in both stress and non-stress conditions	Bouslama and Schapaugh^12^
Harmonic mean	$HM=\frac{2\left(YS\times YP\right)}{YS+YP}$	The genotypes with high HM value will be more tolerant	Bidinger et al.^13^
Geometric mean productivity	$GMP=\sqrt{YP\times YS}$	Higher value of GMP related to high yielding genotypes in both stress and non-stress conditions	Fernandez^14^
Stress tolerance index	$STI=\frac{YS\times YP}{{\left(\overline{Y }P\right)}^{2}}$	Higher value of STI indicates high yield under both stress and non-stress conditions	Fernandez^14^
Yield index	$YI=\frac{YS}{\overline{Y}S }$	The genotypes with high value of YI are suitable for drought stress condition	Gavuzzi et al.^15^

YP and YS: grain yield of a genotype under non-stress and drought stress condition, respectively. $\overline{\mathrm{Y}}\mathrm{P }$ and $\overline{\mathrm{Y}}\mathrm{S }$: average grain yield over all rice genotypes under non-stress and drought stress condition, respectively.

Biplots are used to better understand the correlations among the indices of drought tolerance with grain yield under stress and non-stress. The PCA biplots based on principal component analysis are used. In this regards, we graphically analyzed our data to identify and interpret the best lines in six environments (two conditions of non-stress and drought stress for 3 years) using GGE biplot software^19^. For a better understanding of the relationship among grain yield and indices and the identification of the best lines in each year and in average of the 3 years, GT-biplot analysis^23^ were performed using the genotypes × trait two-way table where the traits include grain yield in non-stress (YP) and drought stress (YS) conditions, and the tolerance and susceptibility indices. The tester-focused scaling was used in visualizing for indices comparison and the symmetrical scaling was utilized in visualizing the which-won-where pattern of lines and years^19^. A scaling method describes the type of standardization used for the mean values before the analysis is performed. Depending on the type of data and the objective of the analysis, GGE biplot uses proper scaling^38^. Calculation of correlation coefficients was performed using the SPSS SPSS version 22, Chicago, IL, USA^40^.

### Introduction of a new composite tolerance index

In this research, a composite tolerance index is developed to identify and determine tolerant lines. This index is a linear combination of significant indices (CSI). The formula of this index is defined as the average of sum of the product of the correlation coefficients of yield under two conditions (non-stress and stress) in significant indices.

If the index had a significant correlation coefficient with grain yield in both conditions, it was considered a significant index.

$${CSI}_{i}=\frac{1}{2}\left({\sum }_{j}^{n}{r}_{YP.{index}_{j}}\times {index}_{ij}+{\sum }_{j}^{n}{r}_{YS.{index}_{j}}\times {index}_{ij}\right)$$

where ${CSI}_{i}$ is the amount of CSI for ith genotype. The r_YP.indexj_ and r_YS.indexj_ represent significant correlation coefficients between index j and grain yield across all genotypes under non-stress and drought stress conditions, respectively. The ${index}_{ij}$ represents the value of the jth index for the ith genotype.

Here, we presented the linear function of CSI based on the correlation results of indices derived from pooled data over these years, which can be extracted from Fig. 2d. Accordingly, considering the four significant indices MP, GMP, HM, and STI, as index _j=1_ to index _j=4_ , the value of CSI_i_ was calculated as follows:

$${CSI}_{i}=\frac{1}{2}[\left({r}_{YP.MP}\times {MP}_{i}\right)+\left({r}_{YP.GMP}\times {GMP}_{i}\right)+\left({r}_{YP.HM}\times {HM}_{i}\right)+\left({r}_{YP.STI}\times {STI}_{i}\right)+\left({r}_{YS.MP}\times {MP}_{i}\right)+\left({r}_{YS.GMP}\times {GMP}_{i}\right)+\left({r}_{YS.HM}\times {HM}_{i}\right)+({r}_{YS.STI}\times {STI}_{i})$$

For example, the calculation of CSI for L53 was as follows:

$${CSI}_{L53}=\frac{1}{2}[\left(0.96\times 4.286\right)+\left(0.85\times 3.553\right)+\left(0.72\times 2.945\right)+\left(0.83\times 0.788\right)+\left(0.79\times 4.286\right)+\left(0.92\times 3.553\right)+\left(0.98\times 2.945\right)+\left(0.91\times 0.788\right)=10.083$$

(Supplementary Table 3 presents the raw pooled data over these years with the indices and CSI calculation formula for rice genotypes). The value of CSI for L53 in Fig. 7 is shown in red. Calculations and drawing graphs of CSI were performed using Microsoft Excel Software 2016.

## Supplementary Information

Supplementary Information 1.

Supplementary Information 2.

Supplementary Information 3.
