# Supplementary material for: Screening of rice drought-tolerant lines by introducing a new composite selection index and competitive with multivariate methods
Source: Sci Rep. 2022 Feb 9;12:2163. doi: 10.1038/s41598-022-06123-9 (PMC8828889; doi:10.1038/s41598-022-06123-9)
Supplement: Supplementary file 1 — Supplementary Information 1. [file 41598_2022_6123_MOESM1_ESM.docx]

**Screening of rice drought-tolerant lines by introducing a new composite selection index and competitive with multivariate methods**

**Atefeh Sabouri^1^*, Ahmad Reza Dadras^2*^, Matin Azari^1^, Abbas Saberi Kouchesfahani^1^, Mehraneh Taslimi^1^, Reza Jalalifar^1^**

1. Department of Agronomy and Plant Breeding, Faculty of Agricultural Sciences, University of Guilan, Rasht, Iran.
2. Crop and Horticultural Science Research Department, Zanjan Agricultural Resources Research and Education Center, Agricultural Research, Education and Extension Organization (AREEO), Zanjan, Iran.

* Corresponding authors address: Atefeh Sabouri: P.O.Box: 41635-1314, IRAN. Tell: +983133690274, Fax: +983133690281. E-mail: [a.sabouri@guilan.ac.ir](mailto:a.sabouri@guilan.ac.ir). ORCID code <https://orcid.org/0000-0002-5831-768X>.

Ahmad Reza Dadras: Email: a.dadras@areeo.ac.ir; a.dadras@yahoo.com ORCID code https:/orcid.org/0000-0001-8591-5813

| 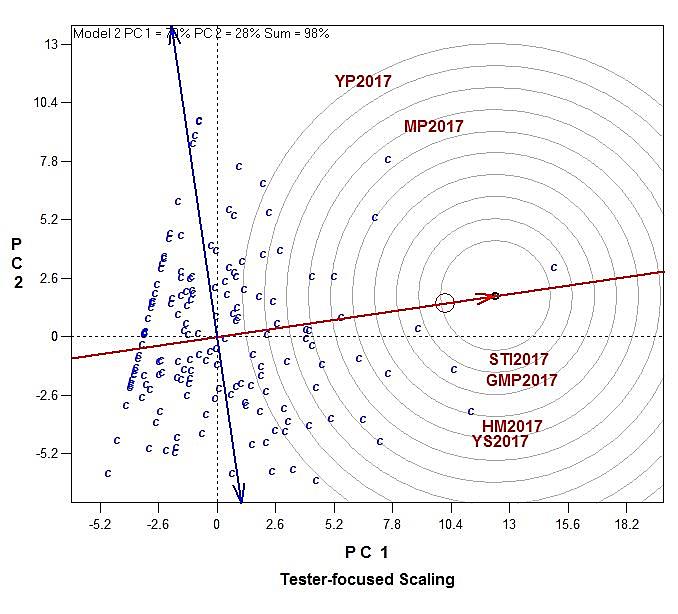  a | 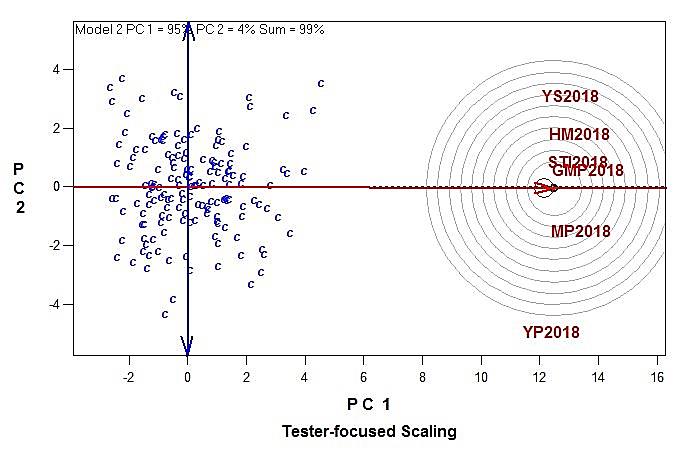  b |
| --- | --- |
| 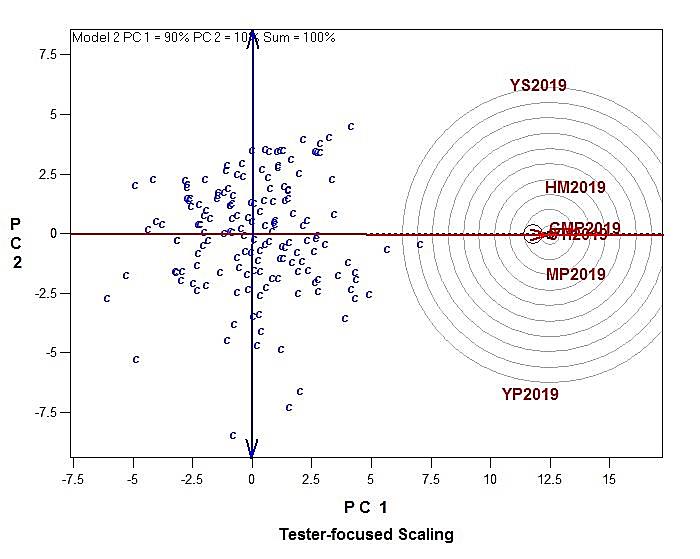  c | 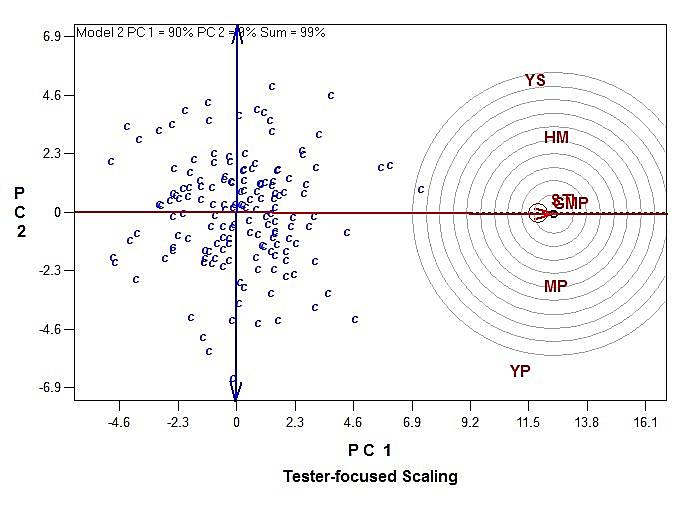  d |

**Supplementary Figure 1.** Comparison of all indices with the ideal index. The ideal index, represented by the small circle with an arrow pointing to it, is the most discriminating of lines and yet representative of the other indices. The indices are ranked based on their distance from the ideal index in 2017 (a), 2018 (b), 2019 (c) and overall mean data of rice lines across three years (d).


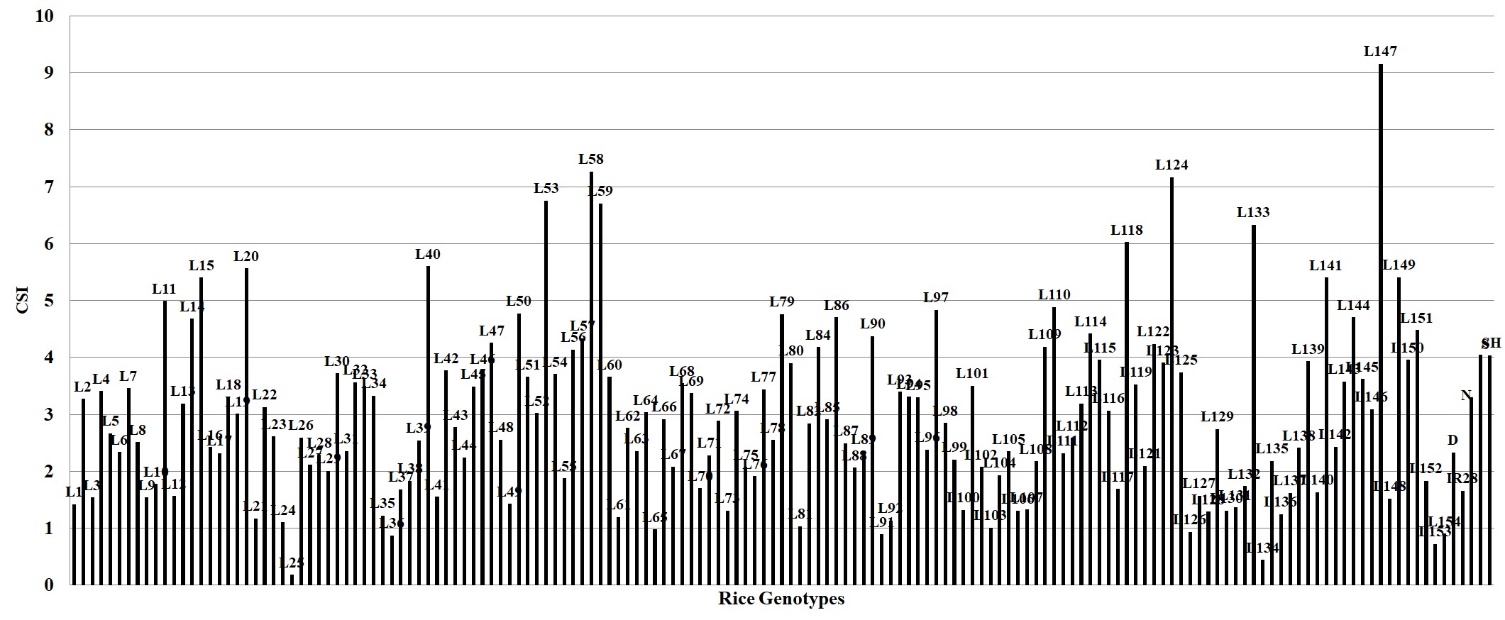


**Supplementary Figure 2.** The CSI value for 152 rice RILs, parental (IR28 and SH: Shahpasand) and check varieties (N; Neda, S; Sadri, D; Dorfak) in 2017.


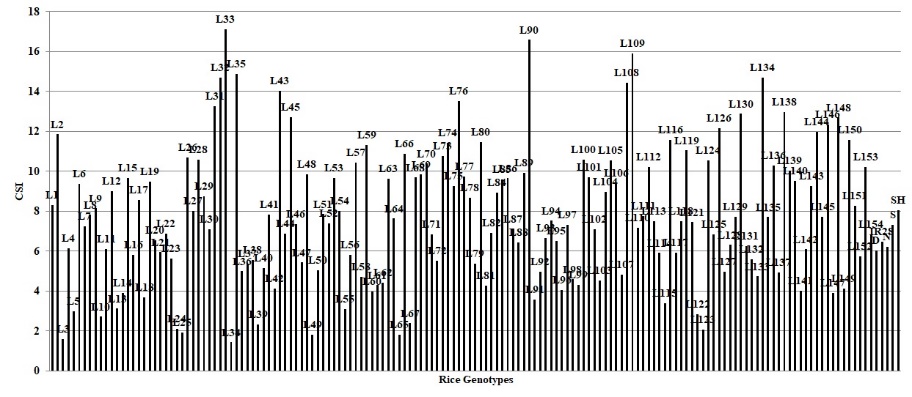


**Supplementary Figure 3.** The CSI value for 152 rice RILs, parental (IR28 and SH: Shahpasand) and check varieties (N; Neda, S; Sadri, D; Dorfak) in 2018.


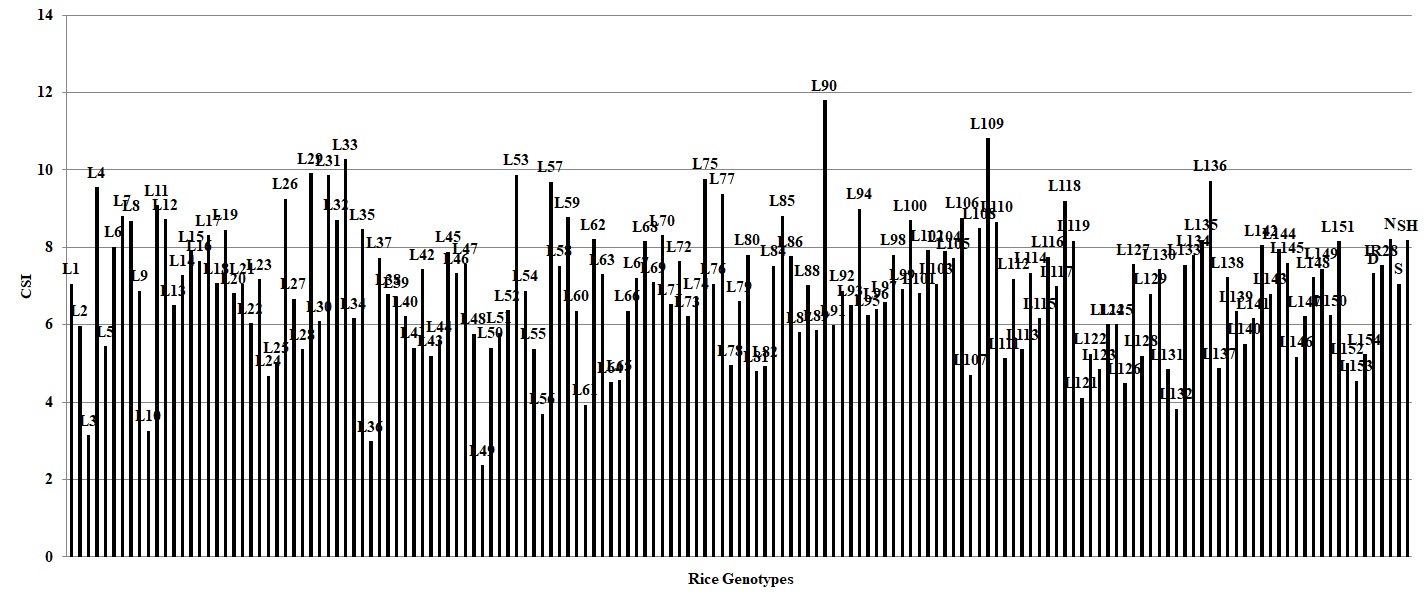


**Supplementary Figure 4.** The CSI value for 152 rice RILs, parental (IR28 and SH: Shahpasand) and check varieties (N; Neda, S; Sadri, D; Dorfak) in 2019.
