# Supplementary material for: Screening of rice drought-tolerant lines by introducing a new composite selection index and competitive with multivariate methods
Source: Sci Rep. 2022 Feb 9;12:2163. doi: 10.1038/s41598-022-06123-9 (PMC8828889; doi:10.1038/s41598-022-06123-9)
Supplement: Supplementary file 3 — Supplementary Information 3. [file 41598_2022_6123_MOESM3_ESM.docx]

**Screening of rice drought-tolerant lines by introducing a new composite selection index and competitive with multivariate methods**

**Atefeh Sabouri^1^*, Ahmad Reza Dadras^2*^, Matin Azari^1^, Abbas Saberi Kouchesfahani^1^, Mehraneh Taslimi^1^, Reza Jalalifar^1^**

1. Department of Agronomy and Plant Breeding, Faculty of Agricultural Sciences, University of Guilan, Rasht, Iran.
2. Crop and Horticultural Science Research Department, Zanjan Agricultural Resources Research and Education Center, Agricultural Research, Education and Extension Organization (AREEO), Zanjan, Iran.

* Corresponding authors address:

Atefeh Sabouri: P.O.Box: 41635-1314, IRAN. Tell: +983133690274, Fax: +983133690281. E-mail: [a.sabouri@guilan.ac.ir](mailto:a.sabouri@guilan.ac.ir). ORCID code <https://orcid.org/0000-0002-5831-768X>.

Ahmad Reza Dadras: Email: [a.dadras@areeo.ac.ir](mailto:a.dadras@areeo.ac.ir);[a.dadras@yahoo](mailto:a.dadras@yahoo).com. ORCID code https:/orcid.org/0000-0001-8591-5813

**Supplementary Table 1.** Meteorological data in Rasht in cropping seasons during 2017-2019

| Month | Year | Sum Rainfall (mm) | Mean tem. (°C) | Mean max tem. (°C) | Mean min tem. (°C) | Mean RH (%) |
| --- | --- | --- | --- | --- | --- | --- |
| Abril | 2017 | 59.00 | 14.20 | 18.48 | 9.92 | 79.63 |
|  | 2018 | 59.24 | 13.68 | 18.95 | 6.60 | 82.07 |
|  | 2019 | 37.11 | 13.60 | 18.31 | 11.20 | 78.20 |
| May | 2017 | 5.90 | 21.30 | 25.66 | 16.95 | 79.19 |
|  | 2018 | 13.61 | 20.79 | 26.25 | 18.70 | 79.81 |
|  | 2019 | 34.40 | 21.60 | 26.80 | 20.30 | 71.56 |
| June | 2017 | 32.40 | 24.46 | 28.73 | 20.19 | 77.32 |
|  | 2018 | 20.30 | 23.88 | 29.25 | 23.00 | 78.39 |
|  | 2019 | 20.50 | 24.88 | 29.30 | 20.10 | 74.13 |
| July | 2017 | 5.00 | 26.83 | 31.65 | 22.01 | 75.03 |
|  | 2018 | 2.40 | 26.43 | 31.95 | 22.60 | 75.69 |
|  | 2019 | 32.62 | 28.99 | 33.38 | 27.80 | 75.81 |
| August | 2017 | 0.00 | 28.24 | 33.61 | 22.86 | 70.90 |
|  | 2018 | 0.01 | 27.83 | 34.01 | 31.00 | 71.93 |
|  | 2019 | 55.00 | 25.78 | 30.13 | 23.10 | 77.29 |
| September | 2017 | 209.90 | 24.57 | 29.06 | 20.08 | 78.63 |
|  | 2018 | 169.11 | 24.13 | 29.52 | 15.30 | 81.74 |
|  | 2019 | 55.40 | 24.07 | 29.32 | 21.00 | 78.37 |
| withholding | 2017 | 0.00 | 27.75 | 32.93 | 21.75 | 72.22 |
|  | 2018 | 58.60 | 27.45 | 32.56 | 26.50 | 73.65 |
|  | 2019 | 68.50 | 27.30 | 32.15 | 24.53 | 76.53 |

**Supplementary** **Table 2**. Statistic descriptive of grain yield under non-stress (YP), and drought stress condition (YS) for 152 rice RILs, parental (IR28 and SH: Shahpasand) and check varieties (N; Neda, S; Sadri, D; Dorfak) across three years (2017-2019).

| Yield (t.ha^-1^) | RILs population | | | | Parental varieties | | | Check varieties | | |
| --- | --- | --- | --- | --- | --- | --- | --- | --- | --- | --- |
|  |  |  |  |  | IR28 | SH | D | | N | S |
|  | Mean | Range | Min | Max | Mean | Mean | Mean | | Mean | Mean |
| YP (2017) | 4.790 | 9.813 | 0.563 | 10.375 | 4.900 | 3.900 | 3.966 | | 6.078 | 3.652 |
| YS (2017) | 0.503 | 2.625 | 0.000 | 2.625 | 0.100 | 1.200 | 0.292 | | 0.333 | 1.304 |
| YP (2018) | 4.080 | 6.048 | 0.945 | 6.993 | 4.543 | 4.072 | 3.630 | | 4.216 | 3.270 |
| YS (2018) | 1.704 | 4.950 | 0.008 | 4.958 | 1.040 | 1.825 | 1.168 | | 1.037 | 1.899 |
| YP (2019) | 3.311 | 3.823 | 1.254 | 5.077 | 3.721 | 3.660 | 3.177 | | 4.154 | 2.853 |
| YS (2019) | 2.033 | 2.969 | 0.442 | 3.411 | 1.986 | 2.352 | 2.216 | | 2.085 | 2.299 |
| Mean YP | 4.000 | 5.246 | 1.438 | 6.684 | 4.388 | 3.877 | 3.591 | | 4.816 | 3.258 |
| Mean YS | 1.413 | 2.481 | 0.294 | 2.775 | 1.042 | 1.792 | 1.225 | | 1.152 | 1.834 |

**Supplementary Table** **4.** Soil water status by sampling the soil every week after water withholding in seven stages throughout the three years.

|  |  | Soil sampling stage | | | | | | |
| --- | --- | --- | --- | --- | --- | --- | --- | --- |
|  |  | 1 | 2 | 3 | 4 | 5 | 6 | 7 |
| 2017 | soil water content (%) | 51.45 | 46.60 | 41.60 | 37.34 | 29.55 | 26.90 | 18.08 |
|  | soil potential (MPa) | -0.02 | -0.04 | -0.10 | -0.21 | -0.79 | -1.25 | -5.63 |
| 2018 | soil water content (%) | 52.92 | 48.57 | 44.11 | 40.75 | 31.31 | 27.96 | 26.31 |
|  | soil potential (MPa) | -0.01 | -0.03 | -0.07 | -0.12 | -0.59 | -1.04 | -1.38 |
| 2019 | soil water content (%) | 54.24 | 48.88 | 45.78 | 43.86 | 40.38 | 31.09 | 27.96 |
|  | soil potential (MPa) | -0.01 | -0.03 | -0.05 | -0.07 | -0.12 | -0.61 | -1.04 |
